# Prevalence of thyroid nodules and characteristics of thyroid ultrasound in children with goiter: a single center experience

**DOI:** 10.1186/s12887-022-03696-2

**Published:** 2022-11-05

**Authors:** Fahad Al Juraibah, Khalid Al Noaim, Abdulaziz AlDbas, Ahmed AlMuallimi, Omar AlOtaibi, Mozon AlShareef, Alanoud AlSuhibani, Ahmed AlZaharani, Mohammed AlDubayee, Amir Babiker

**Affiliations:** 1 Pediatric Department, King Abdulaziz Medical City, King Abdullah Specialist Children’s Hospital, Ministry of National Guard Health Affairs, PO Box 22490 11426, Riyadh, Saudi Arabia; 2 College of Medicine, King Saud bin Abdul-Aziz University for Health Sciences, Ministry of National Guard Health Affairs, Riyadh, Saudi Arabia; 3 King Abdullah International Medical Research Center, Ministry of National Guard Health Affairs, Riyadh, Saudi Arabia; 4 Departments of Pediatrics, College of Medicine, King Faisal University, Al-Ahsa, Saudi Arabia; 5 Department of radiology, King Abdulaziz Medical City, King Abdullah Specialized Children’s Hospital, Ministry of National Guard Health Affairs, Riyadh, Saudi Arabia

**Keywords:** Thyroid nodules, cancer, Hashimoto’s thyroiditis, Ultrasound

## Abstract

**Background:**

Goiter is a common presenting sign of various thyroid diseases in children. Thyroid nodules are clinically and/or radiologically significant findings due to their high malignancy rate. The ultrasound (US) characteristics of pediatric patients with goiter are rarely reported in literature; thus, the purpose of this study is to assess the characteristics of thyroid US and the prevalence of thyroid nodules in pediatric patients with goiter.

**Methods:**

A retrospective review of children and adolescents under the age of 18 (2015–2020) referred for neck ultrasound due to goiter in clinical examination.

**Results:**

A total of 262 patients were included with a mean age of 13.77 ± 3.7 years. Thyroid antibodies were positive in 119/262 (45.4%) patients. Thyroid US reported to be abnormal in 210/262 (80%) patients. Thyroid nodule were found in 33.6% (n = 88/262) of patients with goiter and in 41.9% (n = 88/210) of patients with abnormal thyroid US result. Patients with positive antibodies had more of heterogeneity and hypervascularity of the gland on thyroid US (P < 0.001). On the other hand, thyroid nodules were more likely to be presented in patients with negative thyroid antibodies (P = 0.025). The heterogeneity within the thyroid positive group was significantly correlated with increasing TPOAb (P < 0.001) and TSH levels (P < 0.028). Heterogeneity on US had a positive predictive value (P = 0.041), while hypervascularity had low prediction for thyroid nodules (P = 0.022). Age, gender, family history of thyroid diseases, antibodies status and echogenicity in US did not show any significant associations with thyroid nodules. Papillary thyroid carcinoma was diagnosed in six patients and one of these patients was positive for thyroid antibodies.

**Conclusion:**

Thyroid nodules are quite common in our population. Thyroid nodules were significantly associated with heterogeneity in US. Although, no clinical or biochemical factors could predict the presence of thyroid nodules on thyroid US in our cohort, the absence of thyroid antibodies should lower the threshold for performing thyroid US.

## Background

Thyroid diseases are one of the most common endocrine problems in children and adolescents. In the United States, thyroid disorders are found in 3.7% of children and adolescents between the ages of 11 and 18 years; they also account for 13% of cases that present at endocrine clinics among Saudi adolescents [1, 2]. They usually present for medical attention due to either the presence of an enlarged thyroid gland (goiter), symptoms caused by an excess or deficiency of the thyroid hormone, or incidentally found during a routine biochemistry workup [1]. In Saudi Arabia, the prevalence of goiters in schoolchildren aged 8 to 10 years was found to be < 5% in all regions, except for the Southern region, which has a prevalence of 12.7% [3].

Thyroid ultrasound (US) plays an important role in the evaluation of various thyroid diseases affecting children and adolescents. It is considered the most sensitive imaging tool for evaluating thyroid nodules and thyroid cancer [4]. Preoperatively, US has a sensitivity of 86.5% for diagnosing nonfollicular neoplasm, and 18.2% for follicular neoplasm, and a specificity of 92.3% and 88.7%, respectively [5]. Thyroid US has an important role in detecting focal thyroid disease in children and adolescents with diffuse thyroid goiter [6].

Thyroid nodules are less common in the pediatric population than in the adult population [7]. However, the reported rate of malignancy in these nodules is significantly higher in the pediatric population. Solitary and large nodules, solid parenchyma, a shape that is taller than it is wide, speckled calcifications, irregular margins, and abnormal lymph nodes are all US findings that raise concerns about malignancy and indicate fine needle aspiration (FNA) [8]. Additionally, US findings are reliable in excluding autoimmune thyroiditis (AT); however, they have low sensitivity in distinguishing Hashimoto’s thyroiditis (HT) from Graves’ disease (GD) [9, 10].

AT, including HT and GD, is the most common thyroid disorder in pediatrics [11]. GD is rare compared to HT in pediatrics. HT is more common and can present clinically as a painless, firm, diffuse goiter, often accompanied by hypothyroidism and autoantibodies [11]. However, autoimmune antibodies, namely thyroid peroxidase (TPO) and anti-thyroglobulin (Tg) antibodies, can be absent in 20–50% of patients, highlighting the diagnostic value of thyroid US in evaluating patients who present with goiters [12].

To our knowledge, few local and international reports have addressed the characteristics of thyroid US and its predictive value for diagnosing thyroid nodules in pediatric patients with goiter [13]. We aimed to assess the US features and the prevalence of nodules among children with thyroid goiter in our tertiary center.

## Methods

A retrospective review was conducted using the data of children and adolescents below 18 years of age who underwent neck US from 2015 to 2020 at King Abdullah Specialized Children Hospital (KASCH), Riyadh, Saudi Arabia. Patients referred for neck US due to the presence of a goiter which was discovered during an outpatient visit clinical evaluation were included. We excluded patients referred for neck US for reasons that unrelated to the thyroid gland, patients with Down syndrome with high prevalence of autoimmunity. The clinical information that was collected included age, gender, thyroid antibody status (TPO and Tg antibodies), TSH and free thyroxin (FT4) levels, and US features of the thyroid gland including thyroid gland volume, echogenicity, heterogeneity, vascularity, and presence of nodules. Thyroid US was performed by physicians who qualified and trained in performing neck US in children, and the report was considered abnormal if any of the following were found: presence of thyroid nodules or cysts, abnormal heterogeneity, abnormal size, abnormal echogenicity, or abnormal vascularity. The thyroid glands or nodule was considered to have abnormal vascularity if the vascular flow detected with Doppler US in the thyroid nodule or gland was greater or lower than the surrounding thyroid tissue or the surrounding pre-thyroid muscle (the strap muscle), respectively; and abnormal echogenicity if it appeared hypoechoic or hyperechoic compared to the normal thyroid parenchyma.

Thyroid antibodies, TSH and FT4, were measured using chemiluminescent microparticles immunoassay on the Abbott Architic i2000 immunoassay analyzer. The thyroid antibody status of the patient is considered positive if TPOAb is more than 16 IU/ml and/or the Tg antibody is more than 100 IU/ml as per the references of the laboratory in the study center.

The study was approved by the Institutional Review Board at King Abdullah International Medical Research Center (KAIMRC), Ministry of National Guard Health Affairs, Riyadh, Saudi Arabia (approval no. RC20/214/R). The methods of the study were carried out in accordance with relevant guidelines and regulations including the Declaration of Helsinki for human studies by the World Medical Association. Because of the study’s retrospective nature, King Abdullah International Medical Research Center (KAIMRC) Institutional Review Board specifically waived the requirement to obtain parental or guardian informed consent to conduct this study. The waiver will not adversely affect the rights of the patients and all data relating to patients are kept anonymized.

### Statistical analysis

Data were analyzed using the SPSS 22 (IBM Corp., New York). Continuous variables were expressed as mean ± standard deviation (SD); categorical variables were expressed as percentages. Chi-square tests were used for categorical variables. T-tests and one-way ANOVA were used for continuous variables. Logistic regression was used to assess risk factors. A P-value < 0.05 was considered statistically significant.

## Results

The sample consisted of 262 patients (mean age ± SD 13.77 ± 3.8 years, 84.4% (n = 221) were female). They were referred for thyroid US between 2015 and 2020 due to a clinical finding of goiter. The antibody status was positive in 45.4% (n = 119/262) of patients. The mean TSH level was 8.03 ± 31.62 IU/L (Table 1). US results were abnormal in 80.2% (n = 210/262) of patients while all sonographic parameters were normal in 19.8% (n = 52/262). Thyroid gland was found to be heterogeneous in 74.8% (n = 157/210),homogenous in 20% (n = 42/210), and it was not documented in 5.2% (n = 11/210). In relation to echogenicity, the gland was hypoechoic in 5.7% (n = 12/210), hyperechoic in 1% (n = 2/210), and normal in 93.3% (n = 196/210). In terms of vascularity, the thyroid gland appeared hypervascular, hypovascular, and normal in 64.8% (n = 136/210), 1.4% (n = 3/210) and 33.8% (71/210), respectively. Thyroid nodules were found in 41.9% (n = 88/210) of patients with abnormal US result and in 33.6% (n = 88/262) of patients with goiters. In the regression analysis of 88 patients with thyroid nodules in thyroid US, the likelihood of the presence of nodules was four times greater in heterogeneous glands and significantly lower in hypervascular glands (P = 0.022, OR 0.222, 95% CI: 0.061–0.806)( Table 2).

**Table 1:** Demographic and baseline clinical characteristics of 262 patients with goiter. TSH: thyroid Stimulating Hormone; ND: not done; US: ultrasound

Characteristics	
**Mean age (SD), (min-max) – years**	13.77 (3.8), (2.12–17.9)
**Gender n (%)**	
Male	41 (15.6)
Female	221 (84.4)
**Family history of thyroid disease n (%)**	
Yes	105 (41)
No	155 (59)
**Mean TSH (SD)**	8.03 (31.62)
Thyroid antibodies status n (%)	
Positive	119 (45.4)
Negative	55(21)
ND	88 (33.6)
**Thyroid US overall result**	
Normal	48 (19.80)
Abnormal	210 (80.20)

**Table 2:** Multivariate Logistic Regression (Factors Predicting Presence of thyroid Nodules)

Factor	P-Value	Odd Ratio (95%CI)
Age at Ultrasound	0.117	1.105 (0.975–1.251)
Gender (Female vs. Male)	0.713	1.285 (0.338–4.879)
TSH Level	0.234	1.006 (0.996–1.015)
Family History (positive vs. Negative)	0.95	1.027(0.445–2.37)
Heterogenicity (Heterogenous vs. Homogenous)	0.041*	4.37(1.065–17.93)
Echogenicity (normal is the reference) Hypoechoic	0.612	0.619(0.097–3.938)
Hyperechoic	1	-
Vascularity (normal is the reference) Hyper-vascular	0.022*	0.222(0.061–0.806)
Hypo-vascular	0.609	0.442(0.019–10.116)
Antibodies State (Positive vs. Negative)	0.214	0.494(0.162–1.504)

* Significant at level 0.05

The thyroid antibody status was higher in female patients (Table 3). No significant differences in TSH and FT4 levels were detected among the study groups of different autoimmunity. Levothyroxine treatment was used more frequently in the antibody positive group 61.3% (n = 73/119). With regards to the US characteristics of the thyroid gland, the echogenicity in the US was not significantly different between the groups (P-value 0.427); however, patients with negative antibodies were likelier to have thyroid nodules (P-value 0.025). While, heterogeneous and hypervascular glands on thyroid US were more common in patients with positive thyroid antibodies (P-values < 0.001 and < 0.001, respectively) (Table 3).

**Table 3:** Clinical, biochemical and radiological characteristics associated with antibodies state. TA, thyroid antibodies; ND, not done; TSH, thyroid Stimulating Hormone, FT: free thyroxin

	TA negative (n = 55)	TA positive (n = 119)		P- value
Gender (n, %)				0.018*
Male	13 (23.6)	12 (10.1)		
Female	42 (76.4)	107 (89.9)		
Mean TSH* (SD)	3.88 + 11.75	12.96 + 44.31		0.137
Mean FT4 (S)	13.59 + 3.79	13.49 + 5.12		0.904
**Levothyroxine Usage (n, %)**				< 0.001*
Yes	8 (14.5)	73 (61.3)		
No	47 (85.5)	46 (38.7)		
**Presence of nodule (n, %)** **Yes** **No** **Heterogenicity (n, %)**	24 (43.6%) 31 (56.4%)	31 (26.5%) 86 (73.5%)		0.025* < 0.001*
Homogenous	32 (62.7)	13 (11.2)		
Heterogenous	19 (37.3)	103 (88.8)		
**Echogenicity (n, %)**				0.427
Normal	53 (96.4)	111 (93.3)		
Hypoechoic	1 (1.8)	7 (5.9)		
Hyperechoic	1 (1.8)	1 (0.8)		
**Vascularity (n, %)**				< 0.001*
Normal	23 (63.9)	11 (10.3)		
Hyper-vascular	12 (33.3)	95 (88.8)		
Hypo-vascular	1 (2.8)	1 (0.9)		

*TSH value represent the baseline level before starting levothyroxine

+Significant at level 0.05.

**Fig. 1:**
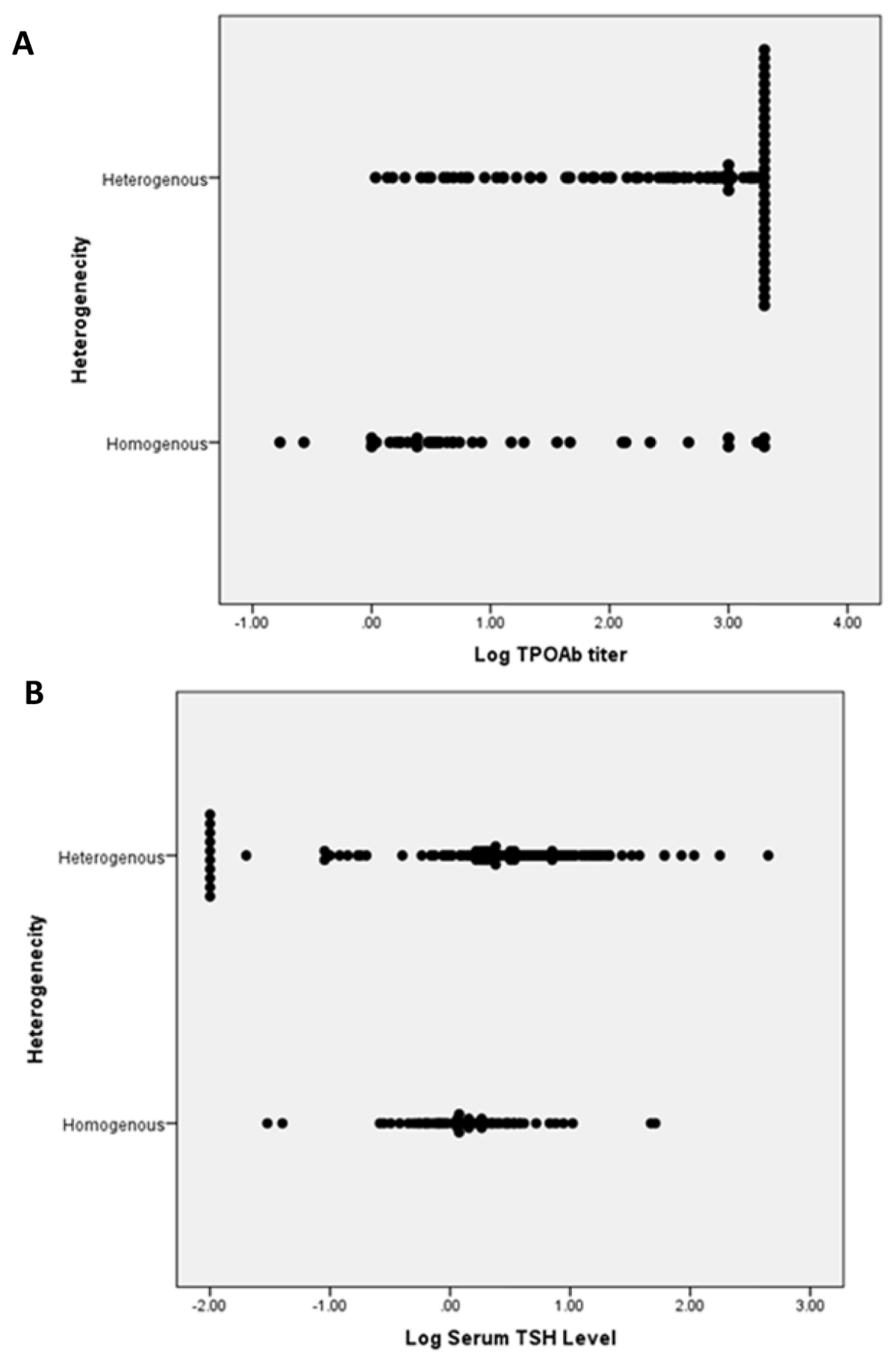
A,B Relationship between presence of heterogenicity of the thyroid gland with log10 TPOAb titer (A) and log10 serum TSH level (B). There was relationship between presence of heterogenecity and log10 TPO titer ( p < 0.001) and log10 serum TSH level ( p = 0.028)

The heterogeneity within the thyroid antibody positive group significantly correlated with increasing TPOAb and TSH levels (P-values < 0.001 and 0.028, respectively). The mean log_10_ TPO was significantly higher in heterogeneous thyroid glands compared to non-heterogenous thyroid glands (2.49 ± 0.95 vs. 0.96 ± 1.1). Also, noted that the mean log_10_ TSH was significantly higher in heterogenous thyroid glands compared to non-heterogeneous thyroid glands (0.41 ± 0.87 vs. 0.17 ± 0.48) (Fig. 1 A and B).

Out of 88 patients who have nodule, 31.8% (n = 28/88) of patients underwent fine needle aspiration (FNA) due to different suspicious reasons. The main indication of FNA was the size of the nodule > 0.5 cm and the presence of other US features of the nodule, such as hypervascularity, hypoechogenicity, presence of microcalcification, or regional lymph node enlargement. 8% of thyroid nodules (n = 7/88) and 25% of FNA samples (n = 7/28) were found to be malignant. Six cases were diagnosed with papillary thyroid cancer (Table 4), and one case diagnosed with medullary thyroid cancer (MTC) who has another family member being diagnosed with MTC. Five of the patients were female, aged from 15 to 17 years. Thyroid antibodies were positive in only one patient. Three of the cases had nodules that were less than one centimeter in diameter. The gland was heterogeneous and/or hypervascular in the majority of the patients in US. Hypoechogenicity and lymph node enlargement were seen in the majority of the patients. The presence of calcification was not frequent.

**Table 4:** Clinical and US Findings of the Patients with Papillary Thyroid Cancer. TN, Thyroid Nodule; TA, Thyroid antibodies; LNE, Lymph Node Enlargement; Pos, positive; Neg, negative; ND, not done

Gender	Age (yrs)	TN size (cm)		TA status		Vascularity		Heterogenecity	Echogenicity			Calcification	LNE			
F	17	2.3		Neg		Hyper-vascular		Yes	Normal			No	No			
F	15	0.6		Pos		Hyper-vascular		Yes	hypoechoic			Yes	No			
F	15	2.5		ND		Hyper-vascular		Yes	Hyperechoic			No	Yes			
M	16	0.6		Neg		Normal		Yes	Normal			No	Yes			
F	16	1.6		Neg		hypervascular		No	hypoechoic			Yes	Yes			
F	17	0.7		Neg		Hypo-vascular		yes	hypoechoic			No	Yes			

## Discussion

Whilst thyroid nodules have been previously reported in 13% of children and adolescents with goiter, the 33% prevalence rate reported in this study is similar to that of another large, multicenter study with thyroid nodules detected in 31.5% of patients with autoimmunity [13, 14]. In our cohort, the prevalence of thyroid nodules was detected in 26.5% of patients with positive thyroid antibodies [14]. The slightly higher prevalence of thyroid nodules in our cohort could be attributed to an increased rate of iodine deficiency among Saudis, which has been reported to affect a quarter of Saudi school-children in a national study, with the southern region of Saudi Arabia being the lowest urinary iodine concentration. [15]. Iodine deficiency has been linked to the incidence of thyroid nodules and other thyroid conditions [16, 17]. As our community has a high rate of consanguinity marriage [18], another explanation for the high frequency of thyroid nodules in our cohort could be related to the genetic predisposition; a family history of various thyroid illnesses was recorded in 41% of our patients, which is a well-known risk factor for thyroid nodules [19]. Our cohort included cases with a clinical diagnosis of goiter, which may further explain the high prevalence of thyroid nodules.

Although thyroid nodules were likelier to occur in patients with negative thyroid antibodies, antibody status did not predict the presence of thyroid nodules in our cohort. Heterogeneity in the US was associated with a higher risk of thyroid nodules. However, age, gender, family history of thyroid diseases, and echogenicity in thyroid US did not show significant associations. Heterogeneity in the US is a common finding in patients with autoimmune thyroiditis [20]. In our cohort, positive antibodies were associated with a high prevalence of heterogeneity, hypervascularity, and normal echogenicity. In addition, heterogeneity was positively associated with TPO and TSH levels. This finding is inconsistent with other reports from previous studies on pediatrics and adults with AT [13, 20]. AT is a potential risk factor for thyroid nodules [14], antibody status was not a contributing factor in our cohort and this could be explained by the fact that patients who were negative for antibodies could have AT with normal antibodies, giving that antibodies fluctuate over time during the course of thyroid disease [21].

Of the patients with thyroid nodules in our data, one third underwent FNA. Papillary thyroid carcinomas were found in six patients and medullary thyroid carcinoma in one patient. Papillary carcinomas mainly affect adolescents between 15 and 17 years. Most of the cases showed normal echogenicity, which were not in line with common hypoechogenic findings in cases of thyroid carcinomas [7]. Various international recommendations have been developed to stratify the risk of thyroid nodules based on US sonographic features [22]. While these recommendations are widely used in the adult population and lead to a reduction in the number of thyroid nodule biopsy, their usefulness in the pediatric population is still debatable and requires further research [23]. The indications for FNA of pediatric thyroid nodules, as recommended by the American Thyroid Association (ATA), are a nodule ≥ 1 cm, history of radiation exposure, or suspicious US features (hypoechogenicity, irregular margins, increased intranodular blood flow, microcalcifications, and enlarged lymph nodes), but the ATA in the last report emphasizes that US characteristics and clinical context, rather than size alone, should be used to identify nodules that require FNA. [7]. Researchers identified that a lack of response to levothyroxine therapy or an increase in nodule diameter during levothyroxine therapy are additional factors that suggest malignancy [14]. The cancer rate in patients with one nodule was higher than that in patients with multiple nodules [9]. On the other hand, nodular HT may demonstrates certain features that correlate strongly with benign pathology, such as a sponge-like appearance, greater than 50% cystic components, hyperechoic echogenicity, and a thin, regular halo [24]. Sonoelastography, a non-invasive technique, can be used to distinguish pseudonodules from true nodules to eliminate unnecessary FNA [25]. Using a standardized reporting system to describe US features of thyroid nodules, such as the American College of Radiology Thyroid Imaging Reporting and Data System, may prevent underdiagnosing thyroid cancer and performing unnecessary workup for patients with benign nodules [26]. In our cohort, FNA-confirmed carcinomas were present in one male (10%) and six females (7.6%). Studies have suggested that a thyroid nodule in a male patient may have a higher rate of malignancy, and a male-to-female ratio of 1:4 for benign nodules was found, but the ratio for malignant nodules was 1:1 [27]. These findings emphasize the importance of thyroid US as a diagnostic modality in cases of goiter, especially in male patients and in cases of negative thyroid antibodies.

US features of nodular HT are variable and there is overlapping of findings commonly seen in benign and malignant nodules [20]. Pediatric HT is commonly associated with enlarged lymph nodes in the lower part of the thyroid gland with 98% sensitivity and 100% specificity [21]. Although FNA is the gold standard for diagnosing HT, it is not used in routine clinical practice because of its invasive nature [24]. HT is generally diagnosed based on clinical data and elevated anti-TPO and/or anti-Tg AB levels. The sensitivity of biomarkers in cases of HT is about 90%, but they can sometimes be negative in histologically proven cases of HT [25]. In Italy, thyroid nodules were found in 31.5% of cases of HT, where thyroid cancer was found in 9.5% of patients with thyroid nodules, and 45% of cancers were associated with lymph node metastasis [26]. FNA was indicated if the nodule size was ≥ 1 cm, which may explain the high incidence of lymph node metastasis in Italian patients [26]. In our study, out of the six cases of papillary carcinoma, three cases (50%) had thyroid nodules less than 1 cm in size. Thyroid nodules in children have a risk of malignancy three to five times greater than the adult population [27]. It is crucial to revise the indicated size for biopsy in pediatric thyroid nodules, and we propose that the decision for FNA be based on a scoring system that includes the clinical background, radiological characteristics, and nodule size, and this requires further studies.

Lastly, the high rate of abnormal US results and nodules in cases of goiter underlines the importance of performing thyroid US in thyroid patients with goiter as a safer approach to our pediatric population. The overlap between benign and malignant nodules and the high prevalence of malignant nodules in the pediatric age group indicates the importance of a low threshold for FNA, regardless of nodule size. We recommend that a thyroid US be performed as a baseline for each patient with goiter, particularly male patients and those with negative antibodies, during the initial visit that needed to be proven by further prospective studies. We also recommend a low FNA threshold in suspicious nodules, regardless of size, which will require further prospective studies. The limitations of this study include being a retrospective and a single center experience. Also, the documented results of thyroid US were written by different radiologists, which may contribute to discrepancies in the thyroid US results.

## Conclusion

Despite the possibility that some cases may have been referred from an area with low levels of iodine, our study found a high risk of thyroid nodules in pediatric patients with goiter. emphasizing the importance of initial screening with thyroid US in these patients to rule out malignant masses. An early diagnosis of thyroid cancer and its appropriate management could be facilitated by adequate clinical and radiological follow-up for patients with HT or thyroid nodules and a low threshold for FNA.

## Data Availability

The datasets generated and/or analyzed during the current study are not publically available due to patient confidentiality but are available from the corresponding author on reasonable request.

## Abbreviations

US: ultrasound
TPOAb: thyroid peroxidase antibody
TSH: thyroid stimulating hormone
AT: autoimmune thyroiditis
HT: hashimoto’s thyroiditis
GD: graves’ disease
TPO: thyroid peroxidase
Tg: thyroglobulin
FT4: free thyroxin
FNA: fine needle aspiration
